# An Insight into the Effect of Exercises on the Prevention of Osteoporosis and Associated Fractures in High-risk Individuals

**DOI:** 10.5041/RMMJ.10325

**Published:** 2018-01-29

**Authors:** Helen Senderovich, Andrew Kosmopoulos

**Affiliations:** 1 Geriatrics & Palliative Care & Pain Medicine, Baycrest Health Sciences, Toronto, Ontario, Canada; 2 Assistant Professor of the University of Toronto, Department of Family and Community Medicine, Division of Palliative Care, Toronto, Ontario, Canada; 3 Research Assistant, Baycrest Health Sciences, Toronto, Ontario, Canada; 4 McMaster University BHSc (Honours), Hamilton, Ontario, Canada

**Keywords:** Bone mineral density, fracture prevention, high-impact exercise, high-intensity progressive resistance training, high-risk population, non-pharmacological treatment for osteoporosis

## Abstract

The purpose of this review was to investigate what type of exercises can potentially prevent osteoporosis (OP) and its associated fractures in high-risk populations. MEDLINE was searched for work relevant to various types of exercises used to prevent osteoporotic fractures in high-risk population, from the year 1995 onwards. Twelve articles were identified, and, from them, four were deemed suitable to the objective. The studies reviewed show that various types of exercise are effective and safe in preventing the onset of OP. For example, high-intensity progressive resistance training (HiPRT) has been shown to increase vertebral height and femoral neck bone mineral density (BMD), in addition to improving functional performance. Additional studies reviewed suggested that bone reabsorption levels may be positively impacted by low-impact exercise, such as walking. This review provides insight into the effectiveness of various types of exercise to combat and possibly prevent OP for high-risk individuals, which include postmenstrual Caucasian females, people with multiple comorbidities, individuals who smoke or consume alcohol, and the frail elderly population. The prevention of OP should reduce both the social (emotional) and economic burdens faced by patients, caregivers, and health-care systems. Moving forward, research that identifies and bridges pharmaceutical treatment and exercise should be conducted, in addition to the comparison of passive versus active forms of exercise to determine which treatment best prevents OP in high-risk populations.

## INTRODUCTION

Osteoporosis (OP) is a disease that affects over 200 million people worldwide,^1^ making it the most common type of bone disease.^2^ When the body begins producing more osteoclasts than osteoblasts at peak bone mass, OP may develop. Osteoporosis is a disease where an individual’s bones are more fragile (less dense), with an increased likelihood to fracture.^2^ Osteoclasts are cells that secrete enzymes and acids which reduce/break down bone tissue by disintegration.^3^ Osteoblast cells make new bone tissue by depositing proteins (e.g. collagen) and minerals (e.g. calcium) throughout the skeletal system.^3^ Once peak bone mass is reached, increased bone reabsorption may be the major pathogenetic factor of OP.^4^ This subsequently decreases an individual’s bone mass, putting them at an increased risk of fractures, primarily of the hip, spine, and distal forearm.^5^ It is important to note that repeated falls put an individual at a higher risk for fractures regardless of their bone mineral density (BMD).^6^

Low BMD can be one of the criteria to define a person as at high risk for OP. Generally, high-risk candidates for OP include females, predominately Caucasian or Asian, who have experienced premature menopause, have a vitamin D deficiency, practice prolonged immobilization, are on systemic steroids, have low dietary calcium intake, and/or are elderly individuals.^5^ Other risk factors that can lead to OP and are independent of BMD include: cigarette smoking, low body weight, and excess alcohol consumption.^5^

Moreover, the social burdens associated with OP, including concern over health and well-being, are not the only costs incurred, as economic strain on health-care systems is also prevalent. In 2010, the cost of OP exceeded $65 billion dollars spanning six continents.^7^ Tarride et al. stated: “Prevention of fractures in high-risk individuals is key to decrease the financial burden of osteoporosis.”^8^

Furthermore, since no cure for OP is currently available, pharmacological treatments help individuals with OP by decelerating the negative effects of bone deterioration. The types of pharmacological treatments vary, although the most common medications to treat patients are bisphosphonate drugs (e.g. alendronic acid and risedronic acid).^9^ Unfortunately, pharmacological treatments with bisphosphonate drugs have potential adverse effects on individuals, the most common being heartburn and an upset stomach.^9^ In addition, atypical femur fractures and osteonecrosis of the jaw are also a concern, although occurring in less than 1% of individuals.^10^ A less common treatment includes hormone replacement therapy (e.g. estrogen replacement therapy), but this is not without side effects. Hormone replacement therapy was found to be associated with a cholelithiasis risk (gallstone formation)^11^ and may increase the risk of heart attacks, strokes, and certain types of cancer.^9^

Overall, this review article focuses on alternatives to pharmacological treatments that could prevent OP from developing, i.e. prevent/reduce the decay of BMD. Specifically, attention is targeted towards numerous variations of exercises that may help to prevent OP and minimize the potential negative side effects of medications. Pharmacological treatments and associated effects on OP are not in the scope of this review. The capacity for exercise to act as protection against decreased BMD may subsequently reduce/remove the emotional hardship on families, as well as the economic burden on health-care systems.

## METHOD

Ovid (MEDLINE, PsychINFO, Embase) was searched for scholarly articles on different types of exercise. This included their role in the prevention of OP and osteoporotic fractures in high-risk populations. The definition of OP given by the US National Library of Medicine^12^ was used when selecting articles for analysis. Studies dating from the year 1995 onwards were accepted for inclusion. The age range targeted was 30–85; such a range promotes comparison in younger adults in relation to a senior population. Additionally, studies targeting both premenopausal and postmenopausal women of any ethnicity, regardless of initial bone mass status, were accepted for inclusion in this review. Furthermore, peer-reviewed randomized control trials (RCTs) and cross-sectional study designs, limited only to English language, were included when selecting papers. Based on the criteria outlined, 12 studies were initially identified as potential candidates, although only four were deemed applicable to the objective and covered a wide range of exercises. Specifically, two studies focused on high-impact exercise and two discussed low-impact exercise. Exercises that require both feet to leave the ground (e.g. running, plyometric exercise, etc.) are considered as high-impact.^13^ Conversely, exercises that require at least one foot to be constantly on the ground (e.g. walking or hiking) are considered low-impact.^13^ In addition, a grey literature search was conducted, using the same search strategies, and including Internet searches of Google, CareSearch, and the Grey Literature Report.

Two investigators assessed the quality of the body of evidence for each key question (“good,” “fair,” or “poor”) using methods developed by the US Preventive Services Task Force (USPSTF), based on the number, quality, and size of studies, the consistency of results between studies, and the directness of evidence.^14^ The studies selected were three RCTs and one cross-sectional design. Finally, these articles were critically analyzed and summarized. A summary of the articles can be found in Table 1.

**Table 1:** Types of Exercise that May Prevent OP and Its Associated Fractures. A Summary Outlining the Results of the Studies Reviewed.

Reference	Study Design and Exercise Duration	Participants	Type of Exercise Performed	Outcomes and Conclusions
Watson et al. (2015)^15^	RCT Duration: Twice weekly for 30 minutes	28 postmenopausal females with low bone mass Age: 66.1±4.8 years	Two groups: Group 1: Brief and supervised HiPRT (includes balancing, weight lifting, and impact loading) Group 2: Low-intensity home-based exercise program	Increased height, improved functional performance, improved femoral neck and lumbar spine BMD in Group 1 compared to Group 2 HiPRT is a “safe and effective exercise therapy” for participants
Wayne et al. (2012)^16^	RCT Duration: 9 months of TC training	86 postmenopausal osteopenic women Age: 45–70 years	TC training	Increase in bone formation markers and femoral neck BMD Suggests longer-duration study to support findings
Kitagawa and Nakahara (2008)^17^	Cross-sectional design Duration: 7 consecutive days	113 postmenopausal women Age: 60–85 years	Low-impact exercise: Walking	Increase in bone stiffness Decrease in urinary DPD Walking may “preserve bone health in elderly women”
Heinonen et al. (1996)^18^	RCT Duration: 3 times per week for 18 months	98 premenopausal, sedentary females Age: 35–45 years	Progressive high-impact exercises	Significant increase in femoral neck BMD Studies required to determine if results are sustained long-term

Organized by date: most recent to oldest.

BMD, bone mineral density; DPD, deoxypyridinoline; HiPRT, high-intensity progressive resistance training; RCT, randomized control trials; TC, T’ai-Chi.

## DISCUSSION

### High-impact Exercise

#### High-impact Loading

The effects of high-impact exercise on osteoporotic fractures, over an 18-month time period, were measured in an RCT study.^18^ Specifically, axial and lower-limb sites were targeted and measured three times: at baseline, at 12 months, and at 18 months. The authors assigned 98 sedentary, premenopausal women aged 35–45 years to a training and a control group. The testing involved participants performing progressive high-impact exercises three times per week. Examples of common high-impact exercises included jogging, dancing, and step aerobics. Results indicated that femoral neck BMD “increased significantly” in the training group (mean 1.6% [95% CI 0.8% to 2.4%]) versus the control group (0.6% [−0.2% to 1.4%], *P*=0.006).^18^ This means that if the study was conducted again using different participants, the results would fall within 0.8% to 2.4% and −0.2% to 1.4% for the training group and the control group, respectively, 95% of the time. Based on the data, it can be inferred that high-impact exercises can “improve skeletal integrity … in premenopausal women.”^18^ The adequate sample size and duration of this study permits the conclusion that high-impact exercise “may help decrease the risk of osteoporotic fractures in later life.”^18^ Nonetheless, the longevity of these benefits past the 18-month duration of this study has not been determined. Additionally, it is unknown whether the difference between groups will continue to be statistically significant.

#### High-intensity Progressive Resistance Training

Close to a decade later, high-impact exercise was evaluated again in another RCT study in the form of high-intensity progressive resistance training (HiPRT). This study tested 28 women (66.1±4.8 years of age) in a Lifting Intervention for Training Muscle and OP Rehabilitation (LIFTMOR) trial, which was to determine the efficacy and safety of HiPRT.^15^ High-intensity progressive resistance training is a form of exercise where the frequency and intensity of the training is altered over time in correspondence to an individual’s personal improvements^19^ and includes activities such as weight lifting (weights used should correspond with current strength level). Participants were randomly subjected to “8 months of twice-weekly 30-min supervised HiPRT and impact loading or a low-intensity home-based exercise program of the same duration and dose.”^15^ Low-intensity exercises programs may consist of slow squats and single leg high knees. The study showed that there was an improvement in vertebral height, functional performance, lumbar spine BMD, and femoral neck BMD, all of which were associated with HiPRT and impact-loading exercises compared to control subjects.^15^

Both high-impact exercise papers reviewed provide evidence that progressive training, specifically HiPRT and impact loading, are safe and effective for high-risk patients, especially postmenopausal women, with low bone mass. This subsequently helps to prevent OP by increasing BMD in weight-bearing areas of the body.

### Low-impact Exercise

#### Walking

In a cross-sectional design study, with 113 postmenopausal Japanese women, walking activity was performed to determine its impact on quantitative ultrasound (QUS) parameters of the calcaneus and bone reabsorption.^17^ During the study, participants walked for seven consecutive days. Afterwards, crucial variables were measured, which included QUS parameters (speed of sound, broadband ultrasound attenuation, and stiffness index [Stiffness] of the calcaneus) and urinary deoxypyridinoline (DPD).^17^ Quantitative ultrasound parameters were used to predict OP fractures. Urinary DPD was tested because it can test the effects of treatments for OP, as it mirrors bone reabsorption.^20^ The study concluded that walking had “significant positive correlations” with stiffness in the calcaneus (*r*=0.258, *P*<0.01). Additionally, the women, aged 60–85 years, showed a “significant decrease” in urinary DPD with walking (*r*=−0.262, *P*<0.01).^17^ These results demonstrate that reduction in bone reabsorption levels may be positively impacted by walking activity (i.e. number of steps taken) while reducing the development of OP. Additional follow-up should be conducted to evaluate the long-term validity of the experiment and to investigate any confounding variables evident during the study. For example, a participant may have been ill during parts of the study, decreasing their commitment to the participation requirements. These confounding variables are much more impactful since the study only lasted for seven days. Nevertheless, the data acquired in the timeframe were positive and significant enough to warrant inclusion in this review.

#### Recreational T’ai-Chi

Efficacy of the Chinese martial art, T’ai-Chi (TC), was tested in a pilot pragmatic randomized trial study.^16^ The study comprised 86 postmenopausal women (aged 45–70) and was conducted for nine months. The women were osteopenic, having a low BMD, but did not have OP. Participants were split into two groups: one group trained in TC and were given usual care (UC), while the other control group were just given UC. Subjects were required to participate in TC for at least one hour, twice each week for the first month, and only one class each week in the subsequent eight months. Additional training included two more sessions of TC per week for the first month, and three times per week for the eight months following, which both could be conducted at home. Over the nine months, the participants trained a minimum of 99.5 hours.^16^ A protocol analysis of the study revealed that participants who completed ≥75% of the training requirements had shown a statistically significant difference. Participants subjected to UC lost approximately 1% (−0.98%) of their femoral neck BMD.^16^ In contrast, participants randomized to the TC per protocol subset group increased their femoral neck BMD (+0.04). The comparison of these two groups yielded a statistically significant *P* value of 0.05 in the area of femoral neck BMD.^16^ Having a moderate statistically significant *P* value allowed for the acceptance of the authors’ hypothesis, part of which expects TC to slow or block bone resorption.^16^ Furthermore, additional outcomes demonstrate that the quality of life in the postmenopausal osteopenic women who performed TC improved and that the exercise helped in “attenuating bone loss.”^16^ The article indicated that TC, provided locally, is promising for “reducing multiple fracture risks” and is “safe.” Limitations to the study include a small sample size and a short experimental duration, relative to the time needed to experience the full-intended benefits of TC. Additionally, the authors stated that “adherence to TC training was lower than expected” which brings up the realization that these results may have arisen by chance and not as a product of TC.^16^ Although these results are in coherence with the other studies reviewed, this pilot experiment sets the stage for future, large-scale research to be conducted to fully investigate TC’s benefits.

## AREAS OF FUTURE STUDY

Despite the fact that various types of exercises have been proven to prevent the decrease in BMD in certain areas of the body, in high-risk individuals, research should be conducted to further support the findings from the papers reviewed. For example, research that compares the efficacy of pharmaceutical treatments versus varying types of exercise in the prevention and/or treatment of OP is warranted. This line of research could give insight into which combinations of pharmaceuticals (e.g. bisphosphonate, estrogens) and types of exercise (e.g. HiPRT, martial arts, walking) yield the greatest results for aging or high-risk individuals. Additionally, this type of research would allow for patient customization in OP prevention methods, depending on the individual’s preferences and physical condition. Furthermore, with regard to studies focusing solely on exercise and associated effects on OP, evaluation should be made of various subsets of the type (TC, walking, HiPRT, etc.), frequency, and duration of exercises performed, with a longer follow-up and a larger sample size. This is to investigate and test the results of the articles reviewed, over a longer term with a larger population. Although the results presented were significant, some studies had a small sample size and short follow-up in comparison to others, therefore further investigation of these results would be valuable. Lastly, research in relation to the diagnosis of OP is crucially needed. Although BMD scans assess the risk of developing OP, the disease has minimal symptoms which usually appear when it is too late to intervene effectively. If OP could be diagnosed earlier, the exploration of available treatments may be performed sooner, better protecting an individual from the disease. Lastly, it should be noted that exercise carries a small risk of increasing fractures among individuals at high risk.^6^

## CONCLUSION

Osteoporosis currently affects millions of people worldwide, imposing physical strain in addition to social and economic burdens. The decrease in BMD can lead to fractures (e.g. wrist, lumbar spine and femoral neck regions). Although pharmacological treatments do exist in an attempt to try and decelerate the consequences of low BMD, the side effects associated with these medications, and their failure rate, create a need for an alternative method. In this review, four studies were critically analyzed, giving a comprehensive and thorough summary of a wide range of exercises, which could be the potential alternative to pharmacological treatments. The exercises can be easily performed by at-risk individuals and may assist in the prevention of OP, although caution should be exercised among individuals at high risk. In the studies reviewed, exercise is primarily shown to reduce the loss of BMD and increase bone stiffness, demonstrating its ability to act as a barrier to the development of OP. Specifically, high-impact loading was found to increase femoral neck BMD. High-intensity progressive resistance training may improve vertebral height as well as lumbar spine and femoral neck BMD. In addition, walking was positively correlated with stiffness in the calcaneus and helped to decrease urinary DPD, which may help reduce bone reabsorption. Lastly, performing TC may be beneficial in attenuating bone loss. The findings of the articles are promising with regard to the prevention of OP. The need for consistent exercise both prior to the onset of OP and after the development of the disease is crucial. Exercise may prevent BMD levels from decreasing and could help in OP prevention. These results must be further tested in future experiments as limitations on the duration and length of studies reviewed justify additional research. To conclude, patients currently diagnosed with OP should be closely monitored to determine treatment efficacy for optimal results. Treatment options that are available to them and can be appropriate to their specific situation need to be discussed at the time of the diagnosis.

## Abbreviations

BMD: bone mineral density
DPD: deoxypyridinoline
HiPRT: high-intensity progressive resistance training
OP: osteoporosis
QUS: quantitative ultrasound
RCT: randomized control trial
TC: T’ai-Chi
UC: usual care
